# Radiopathological correlations of myopericytomas of the hand: emphasis on the MRI perivascular pushing growth pattern

**DOI:** 10.1259/bjrcr.20190074

**Published:** 2020-09-29

**Authors:** Amandine Crombé, Amine Bouhamama, François Le Loarer, Michèle Kind

**Affiliations:** 1 Department of Radiology, Institut Bergonié, Comprehensive Cancer Center of Aquitaine, F-33000, Bordeaux, France; 2 Department of Radiology, Centre Léon Bérard, Comprehensive Cancer Center of Rhone Alpes, F-33000, Bordeaux, France; 3 Department of Pathology, Institut Bergonié, Comprehensive Cancer Center of Aquitaine, F-33000, Bordeaux, France

## Abstract

Myopericytomas are exceedingly rare soft-tissue tumors with less than 10 cases including radiological depictions. We report three new cases of benign myopericytomas located in the soft-tissues of the hand in adult patients. A pre-treatment MRI was available for all patients and systematically evidenced well-defined, lobulated tumors closely related to the superficial palmar vascular arch and/or digital vessels with a perivascular pushing growth pattern that correlated with pathological findings. Though rare, this small case series show that myopericytomas display recurrent imaging features that could support their radiological diagnosis.

## Clinical presentation

A 72-year-old male without significant medical history presented with a lump of the right palmar hand side. This lesion was slowly growing for more than 5 years and became progressively painful.

## Imaging findings

**Figure 1.**
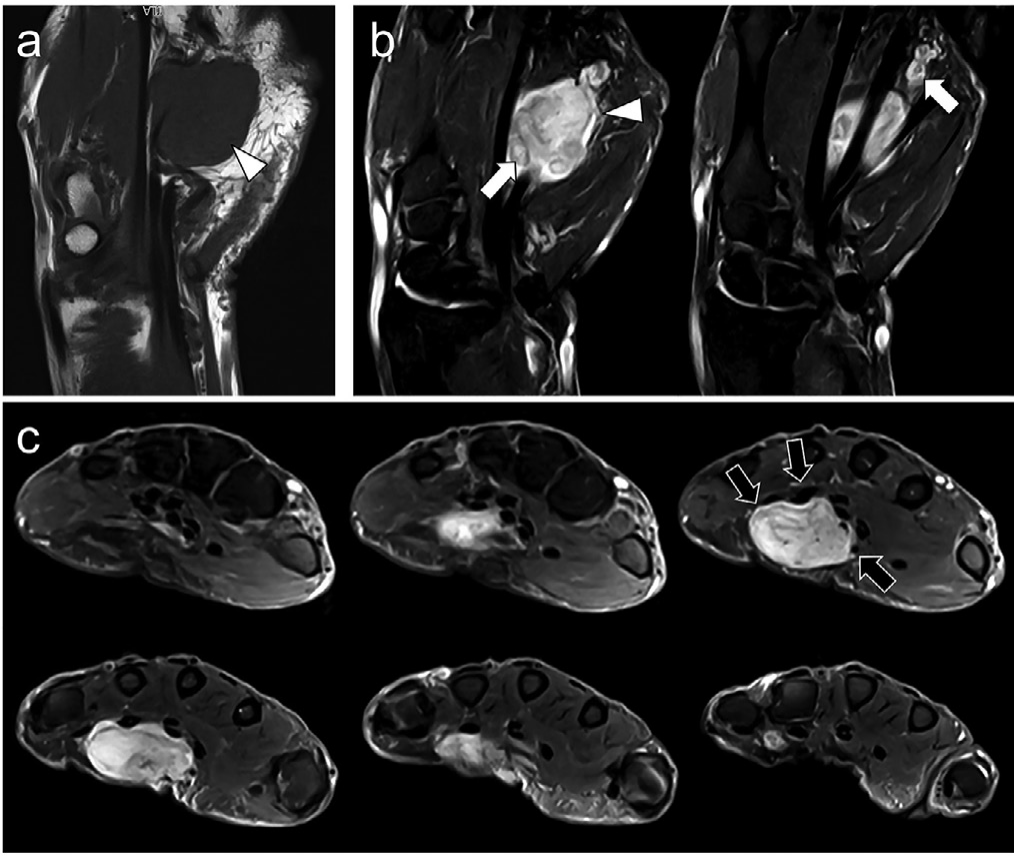
A 72-year-old male with a swelling of the ulnar palmar side of the hand underwent a conventional MRI. (a) Coronal *T*_1_WI showed a 54 mm-long, well-defined, non-encapsulated single tumor with SI similar to normal muscle, without fat or hemorrhage (white arrowhead). (b) Coronal fat-suppressed *T*_2_WI demonstrated high SI and intratumoral nodule delineated by thin strand of lower SI on *T*_2_WIWI (white arrows) leading to a slight heterogeneity. (c) On consecutive axial fat-suppressed *T*_2_WIWI, the tumor demonstrated a close relationship to superficial digital flexor tendons as well as superficial palmar arch with extensions following the third, fourth and fifth fingers. There was no surrounding edema or bone invasion. SI, signal intensity; *T*_1_WI, *T*_1 _weighted imaging;*T*_2_WI, *T*_2 _weighted imaging.

On MRI, the tumor measured 54 mm and was well-circumscribed with lobulated margins. It was closely related to the superficial palmar vascular arch with perivascular nodular clusters demonstrating a pushing growth pattern. The tumor showed thinner extensions following the common palmar digital vessels of the fifth finger (Figure 1). It displayed high signal intensities (SIs) on fat sat *T*_2_ weighted imaging (*T*_2_WI) and low SI on *T*_1_WI, similar to the muscles. There was no cystic, fatty, necrotic, myxoid, or fibrotic component. The surrounding tissues did not exhibit edema, aponeurotic or peritumoral enhancement. The tumor showed a limited intratumoral heterogeneity on *T*_2_WI due to intratumoral nodules delineated by lower SI on *T*_2_WI.

## Differential diagnoses

The close relationship to palmar vessels raised the hypothesis of a tumor of vascular or pericytic origin such as hemangiopericytoma, angioleiomyoma, myofibroma or myopericytoma. The slow progression, the well-defined margins, the lack of necrosis and the limited heterogeneity were rather in favor of a benign lesion but soft-tissue sarcoma, especially leiomyosarcoma, could not be excluded.

## Investigations

An ultrasonography-guided core needle microbiopsy was performed because the tumor was deep-seated, over 5 cm without obvious diagnosis. The histopathological analysis revealed a proliferation of cells containing significant eosinophilic cytoplasm and arranged in short fascicles circumscribing tightly small blood vessels. There was no cytologic atypia and the proliferative activity was low (1–2% of cells positive for Ki67). Immunohistochemistry demonstrated a strong and diffuse positive staining for alpha-smooth muscle actin, desmin and h-caldesmon. These features were compatible with a benign myopericytoma of the hand.

**Figure 2.**
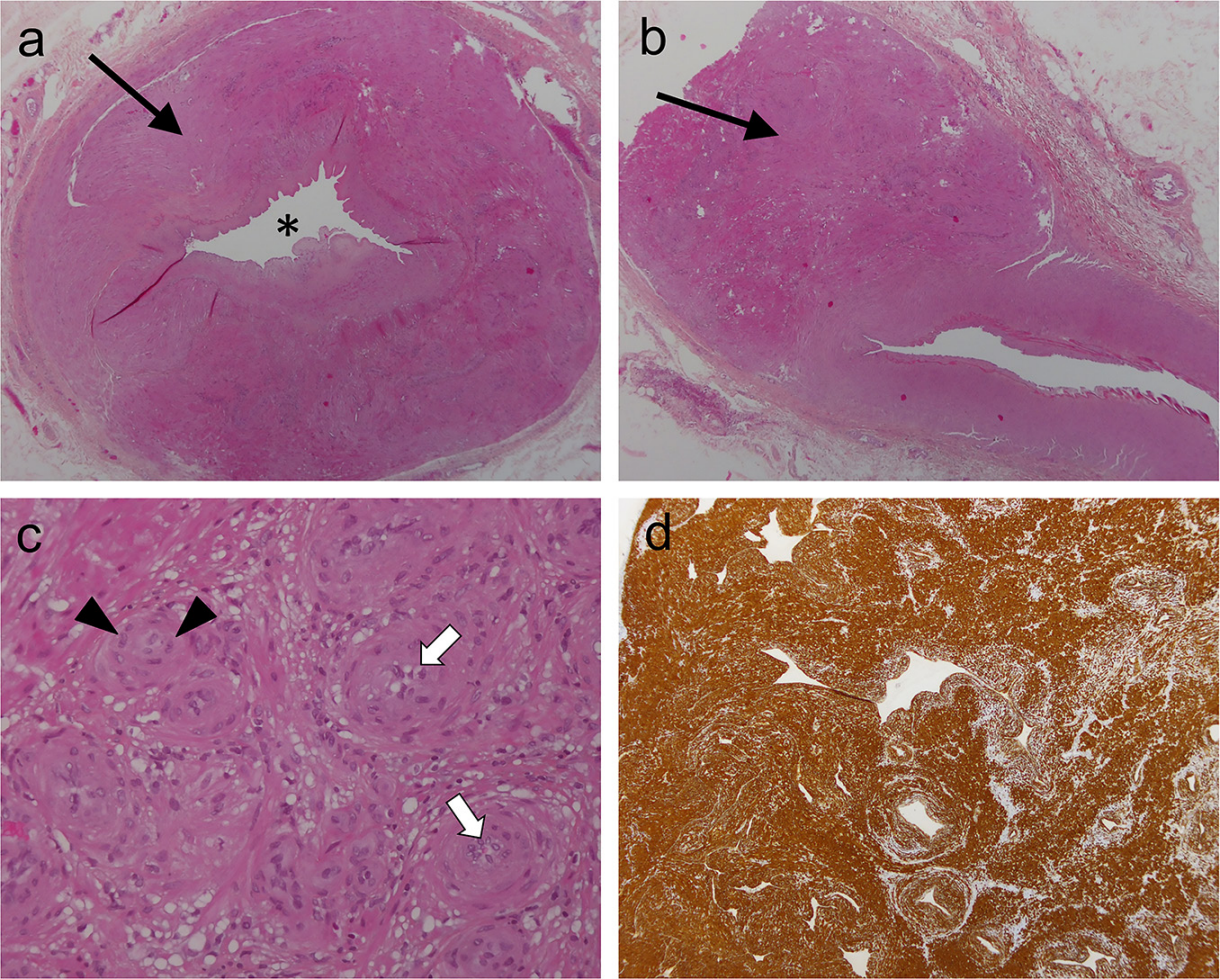
Histopathological analysis of the surgical specimen. (a) HES slice showed a marked concentric thickening of the ulnar side of the superficial palmar arch leading to a narrowing of the vascular lumen (black asteroid), together with (b) perivascular tumor nodule (black arrows). (c) Magnification emphasized the presence of multiple uniform fusiform small cells with eosinophilic cytoplasm inside the vessel wall (white arrowhead), which were organized around smaller vessels (white arrows). (d) Immunohistochemistry demonstrated a strong and diffuse positive staining for h-caldesmon. HES, hematoxylin andeosin stained.

The patient was subsequently treated with a curative surgery. The gross examination of the surgical specimen showed strong analogy with MRI. The tumor was well-circumscribed, multinodular and solid with a yellow-white pearl color. The ‘hemangiopericytic’ concentric perivascular growth pattern was responsible for a thinning of the vessel lumen (Figure 2). No infarction, hemorrhage or necrosis was reported.

## Complementary cases

Following these findings, we performed a request on the pathological databases of two French Sarcoma Reference Centres and we identified a total of 13 patients with myopericytomas of the upper limb including two with a pre-treatment MRI. Interestingly, they also shared this perivascular pushing growth pattern.

**Figure 3.**
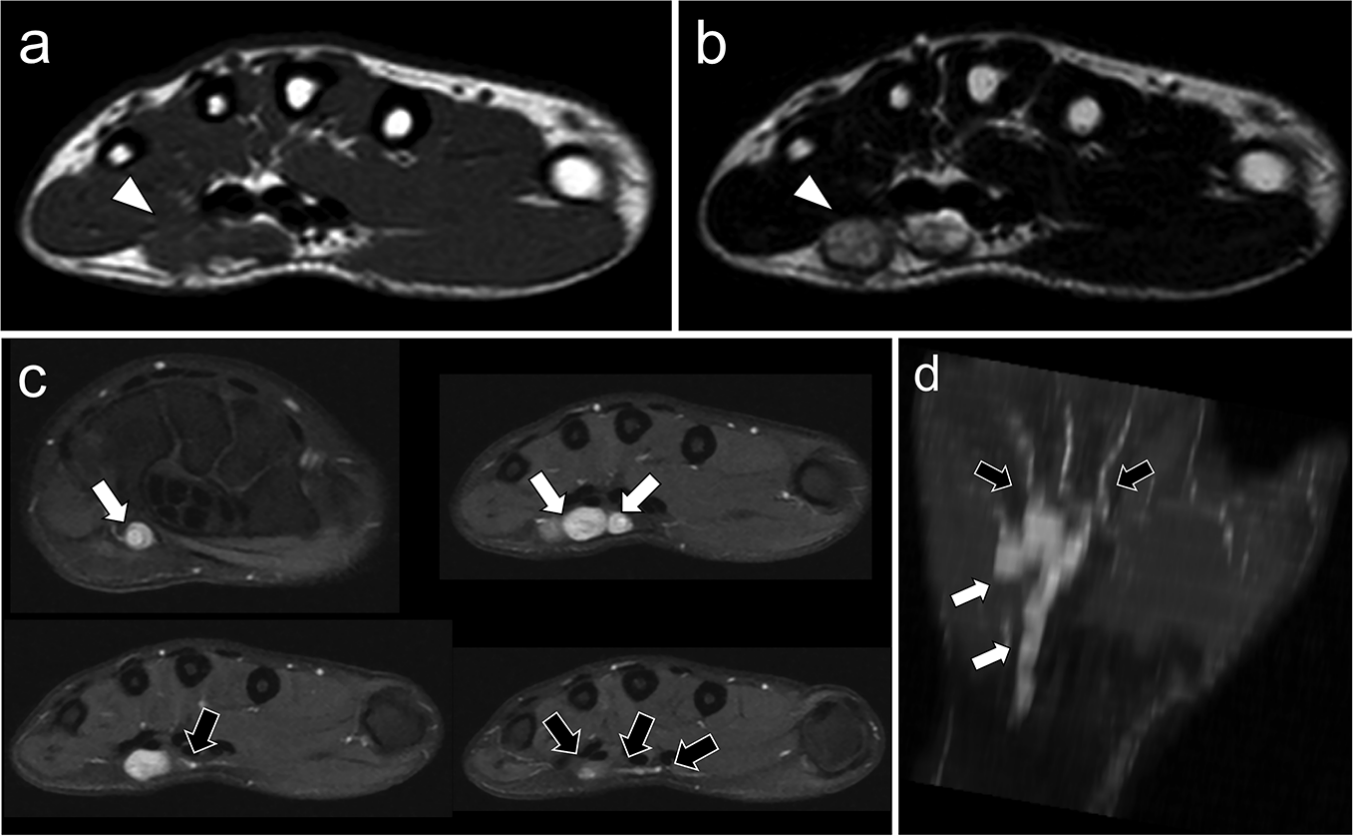
A 30-year-old underwent a contrast-enhanced MRI to characterize a chronic swelling of the palmar and ulnar side of the left wrist. MRI showed a well-defined multinodular 21 mm-large and 45 mm-long tumor (white arrowheads). (a) The lesion had same signal intensities as normal muscle on Axial *T*_1_WI without fat or hemorrhage (white arrowhead). (b) On axial *T*_2_WI, it showed high SI and a moderate intratumoral heterogeneity (white arrowhead). (c) On consecutive axial fat-suppressed, contrast-enhanced *T*_1_WI, the tumor demonstrated a close relationship to ulnar vessels with contiguous nodules spreading along superficial palmar arch (white arrows) and common palmar digital vessels (black arrows). It displayed a marked, homogeneous enhancement, no necrosis. There was no peritumoral enhancement. (d) The coronal reformation of the contrast-enhanced *T*_1_WI clearly illustrates the perivascular growth pattern, with a lobulated tumor spreading along the palmar arch (white arrows) and its digital branches (black arrows). SI, signal intensity; *T*_1_WI, *T*_1 _weighted imaging;*T*_2_WI, *T*_2 _weighted imaging.

The first patient was a 30-year-old female. She presented with a discrete lump of the palmar and ulnar side of the left wrist that became progressively painful with paraesthesia in the ulnar nerve territory. A MRI was performed showing a 45-mm-long, well-circumscribed, lobulated and multinodular tumor that followed the same orientation as the ulnar vessels and spread along the superficial palmar vascular arch with small, thin extensions following the common palmar digital vessels towards the fifth, fourth and third fingers (Figure 3). After intravenous Gadolinium chelates injection, the tumor demonstrated a homogeneous and marked contrast enhancement.

**Figure 4.**
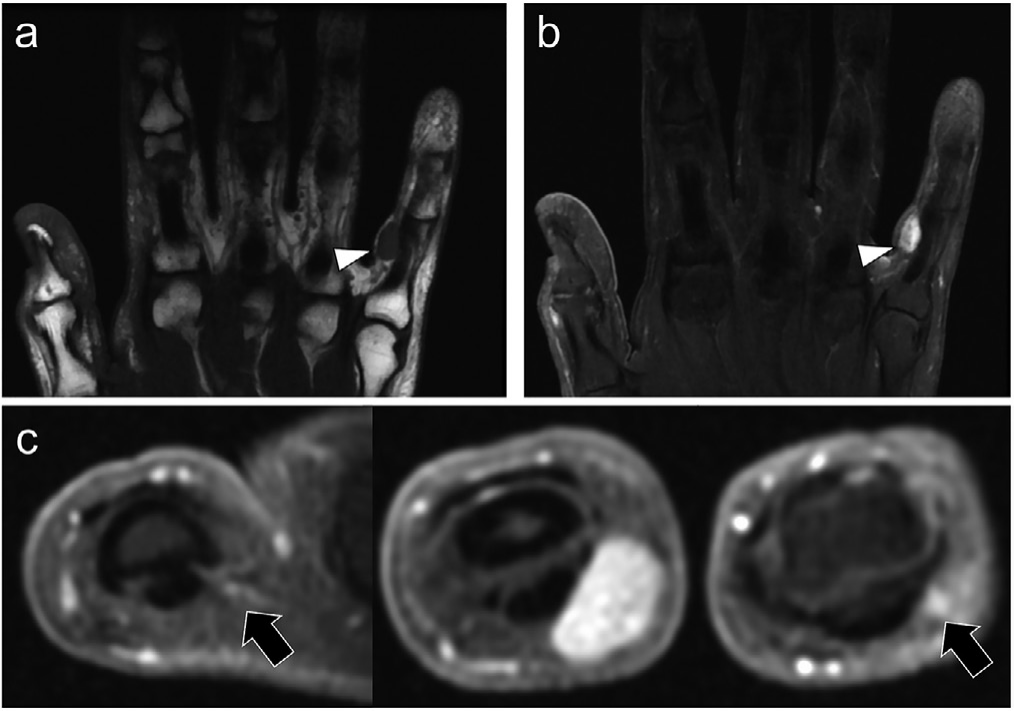
A 51-year-old male underwent a MRI because of a swelling of the fifth finger of the right hand. (a) On coronal *T*_1_WI, the lesion was located on the radial side of the fifth finger. It was 9 mm-long, unique, well-defined, homogeneous and it demonstrated same SIs as normal muscle (white arrowhead). (b) On coronal fat-suppressed *T*_2_WI, the tumor demonstrated homogeneous high SI, without peritumoral edema (white arrowhead). (c) On consecutive axial fat-suppressed, contrast-enhanced *T*_1_WI, the tumor demonstrated marked, homogeneous enhancement, without peritumoral enhancement. It was located in the prolongation of the fifth proper palmar digital vessel (black arrows). SI, signal intensity; *T*_1_WI, *T*_1 _weighted imaging;*T*_2_WI, *T*_2 _weighted imaging.

The second patient was a 51-year-old male who presented with a slowly progressive lump in the fifth finger of the right hand. A MRI was performed before excisional surgery, which demonstrated a 9 mm-long, well-circumscribed, homogeneous, nodular tumor with iso-SI on *T*_1_WI, high SI on fat sat *T*_2_WI and homogeneous contrast-enhancement (Figure 4). The tumor was superficially seated in the soft-tissue of the radial side of fifth finger and developed along the proper palmar digital vessel. A glomic tumor was initially hypothesized because of the close relationship to a digital vessel and the homogeneous enhancement, but the histopathological analysis of the surgical specimen confirmed a myopericytoma.

## Follow-up and outcome

The three patients were treated with surgery only. There was a follow-up of 3, 9 and 7 years respectively, without local or distant relapse according to clinical examination.

## Discussion

Myopericytomas are mesenchymal tumors with both vascular and smooth muscle cell differentiations originating from pericytes, which are perivascular cells adjacent to capillaries.^1^ Myopericytomas were initially individualized in 1996 and further characterized by Granter et al in 1998.^2^ They share histological features with myofibroma and glomic tumors, and partial morphological and histological overlaps can be seen with these other tumors.^1^ Myopericytomas mostly involve the soft-tissues of distal extremities, followed by proximal extremities, head, trunk and oral cavity in adults. The radiological presentation of myopericytomas is poorly known, presumably due to their small size and superficial location leading in many cases to excisional biopsy without pre-operative MRI.

According to case reports, myopericytomas seem to occur slightly more often in males, from 13 to 75 years old. Their risk factors are unclear but some case reports have highlighted a context of prior trauma or immunodeficiency.^3,4^ Myoperictyomas are generally solitary tumors, even if rare multifocal cases have been found.

Regarding the distal upper extremities, less than 25 cases have been published so far, including less than 10 with imaging. However, they share common features that could help to establish diagnosis.^5–7^ The medical history usually consisted of a slowly progressive painless lump, known for years, and that became inconvenient for daily activities. On ultrasonography, myopericytomas typically demonstrated a hypoechoeic, firm, slightly heterogeneous and well-defined lesion adjacent to vessels, mostly of the palmar side of the hand, with hypervascularity on doppler. Herein, ultrasonography (not shown) was only available for Patient 2 in order to guide the biopsy—the other ones being performed out of our sarcoma reference center and not available on our PACS.

MRI is the best imaging modality for characterizing myopericytomas. In all reports, myopericytomas displayed iso- to low SI on *T*_1_WI, high SI on *T*_2_WI, with a discrete heterogeneity due to an intratumoral nodular aspect, and rather lobulated contour. The sizes ranged from 9 to 54 mm. The previous studies systematically stressed the proximity to an artery or a vein. Interestingly, myopericytomas seemed to encase and push adjacent organs rather than invade them. After contrast agent injection, myopericytomas constantly demonstrated a strong, avid enhancement related to their vascular origin, without peritumoral enhancement. Interestingly, Van Camp et al performed an arteriography and a dynamic-contrast enhanced MRI of a myopericytoma showing an arterial enhancement and a washout.^7^ Such a kinetic of enhancement may be reminiscent of a glomus tumor and could help making diagnosis.

**Figure 5.**
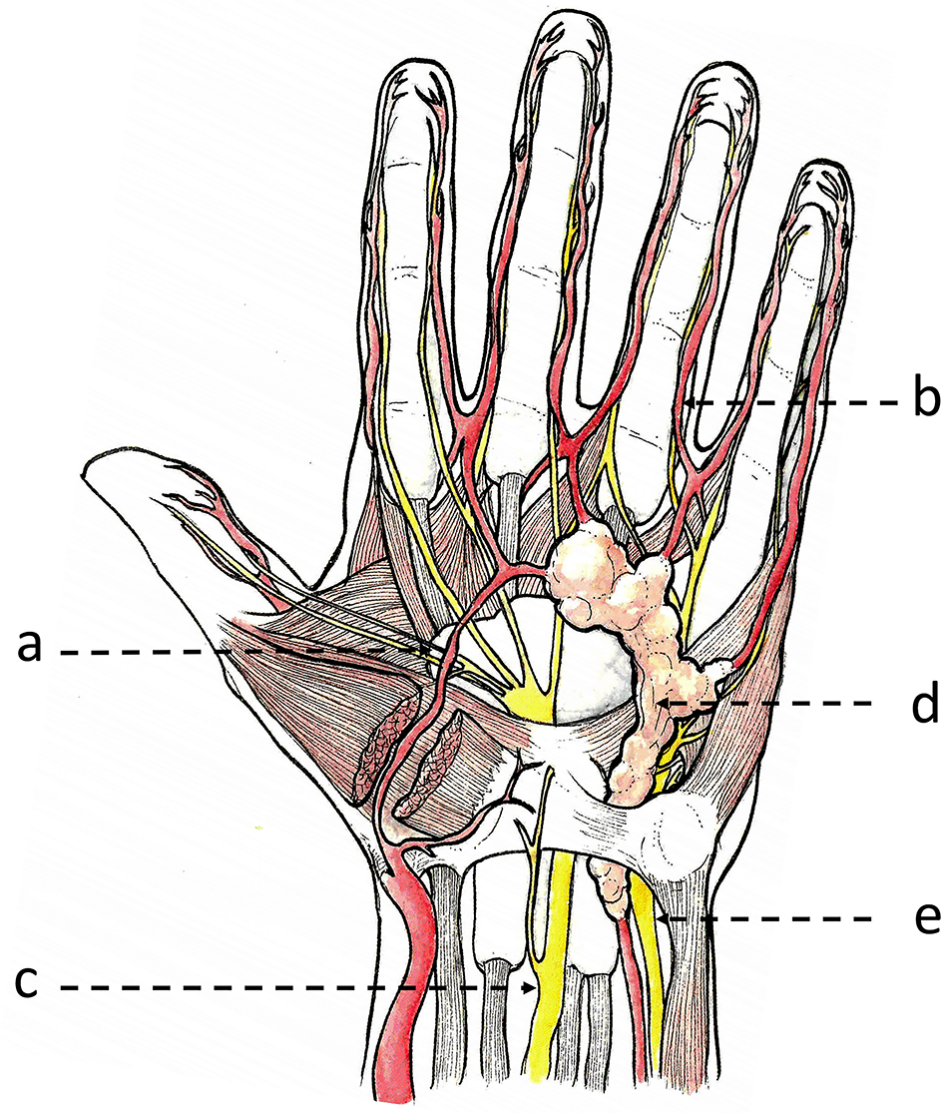
Schematic coronal view of a typical myopericytoma of the hand, which originated from the wall of the palmar vascular arch and has developed along this vessel and its digital branches, with a nodular perivascular pushing growth pattern. Legends: (a) palmar vascular arch; (b) digital vessels; (c) median nerve; (d) myopericytoma; (e) ulnar nerve.

In the three present cases, the final diagnosis was rendered by histopathological analysis. Hematoxylin and eosin stained slides demonstrated the typical features associated with myopericytomas and notably a concentric perivascular growth pattern. This appearance is in keeping with the pericytic origin of these tumors encasing the vascular structure from which they arise and sometimes protruding into the lumens of the vascular branches (Figure 5).^1,2,8^ Immunohistochemistry systematically found strong and diffuse positive staining for ASMA and h-caldesmon, while staining for CD34 and desmin seemed more inconstant.^8^ Interestingly, myopericytomas are underlined by somatic mutations of *PDGFRB* (Platelet derived growth factor receptor B—a growth factor involved in angiogenesis) in about 60% of cases. Nevertheless, it cannot help to distinguish them from other pericytic tumors because PDGFRB alterations can also be found in angioleiomyomas and myofibromas.^9,10^

To the best of our knowledge, 11 cases of malignant myopericytomas have been reported. They were histopathologically characterized by high mitotic rate, hypercellularity, pleomorphism and necrosis but they lacked radiopathological correlations.^11^ Six of these patients developed metastasis in the liver, skin, heart, brain and bone. Three of them died of their disease.

The treatment for benign myopericytomas consists of a curative surgery with rare local recurrence likely due to incomplete resection, thereby requiring regular follow-up based on clinical examination.

To conclude, myopericytomas belong to a continuous spectrum of tumors of pericytic lineage with nodular, concentric, perivascular pushing growth pattern that parallels their imaging features. Myopericytomas of the hand typically present as well-circumscribed and nodular lesions attached to (or encasing) the superficial palmar arch and its digital branches, together with an avid contrast-enhancement on MRI. Patients should be referred to a sarcoma reference center before surgery in order to perform imaging-guided core-needle biopsy because of potential malignancy.

## Learning points

1. Myopericytomas are extremely rare ubiquitous tumours that have been recently described and are mostly encountered in the distal extremities of adults.
2. They share common MRI features that correlate with histopathological analysis. They present as well-limited lobulated and nodular tumors with a close relationship to palmar vessels reminiscent of their pericytic lineage. They rather encase and push aside the vascular and other surrounding structures, instead of invading them.
3. Patients with a suspicion of myopericytoma must be referred to a Sarcoma Reference Center because of potential malignancy.

## References

[1] Fletcher CDM, Bridge JA, Hogendoorn PCW, Mertens F; IARC Press WHO Classification of Tumours of Soft Tissue and Bone. 4th edn Vol 5 Lyon, France; 2013.

[2] Granter SR, Badizadegan K, Fletcher CD Myofibromatosis in adults, glomangiopericytoma, and myopericytoma: a spectrum of tumors showing perivascular myoid differentiation. Am J Surg Pathol 1998; 22: 513–25. 9591720 10.1097/00000478-199805000-00001

[3] Laga AC, Tajirian AL, Islam MN, Bhattacharyya I, Cohen DM, Plamondon CJ, et al Myopericytoma: report of two cases associated with trauma. J Cutan Pathol 2008; 35: 866–70. doi: 10.1111/j.1600-0560.2007.00910.x 18494828

[4] PPL L, Wong O-K, PCW L, Cheung O-Y, L-C H, Wong W-C, et al Myopericytoma in patients with AIDS: a new class of Epstein-Barr virus-associated tumor. Am J Surg Pathol 2009; 33: 1666–72. 19675451 10.1097/PAS.0b013e3181aec307

[5] Kara A, Keskinbora M, Kayaalp ME, Şeker A, Erdil M, Bülbül M An atypical presentation of myopericytoma in palmar arch and review of the literature. Case Rep Orthop 2014; 2014: 1–3. doi: 10.1155/2014/759329 PMC4202198 25349759

[6] Wagner ER, Shin AY Myopericytoma of the hypothenar eminence: case report. HAND 2015; 10: 349–52. doi: 10.1007/s11552-014-9655-5 26034459 PMC4447665

[7] Van Camp L, Goubau J, Van den Berghe I, Mermuys K Myopericytoma of the base of the finger: radiological and pathological description of a rare benign entity. J Hand Surg Am 2019; 44: 69.e1–69.e5. doi: 10.1016/j.jhsa.2018.03.015 29678425

[8] Mentzel T, Dei Tos AP, Sapi Z, Kutzner H Myopericytoma of skin and soft tissues: clinicopathologic and immunohistochemical study of 54 cases. Am J Surg Pathol 2006; 30: 104–13. 16330949 10.1097/01.pas.0000178091.54147.b1

[9] Hung YP, Fletcher CDM Myopericytomatosis: clinicopathologic analysis of 11 cases with molecular identification of recurrent PDGFRB alterations in Myopericytomatosis and Myopericytoma. Am J Surg Pathol 2017; 41: 1034–44. 28505006 10.1097/PAS.0000000000000862

[10] Agaimy A, Bieg M, Michal M, Geddert H, Märkl B, Seitz J, et al Recurrent somatic PDGFRB mutations in sporadic Infantile/Solitary adult Myofibromas but not in Angioleiomyomas and Myopericytomas. Am J Surg Pathol 2017; 41: 195–203. doi: 10.1097/PAS.0000000000000752 27776010

[11] McMenamin ME, Fletcher CDM Malignant myopericytoma: expanding the spectrum of tumours with myopericytic differentiation. Histopathology 2002; 41: 450–60. doi: 10.1046/j.1365-2559.2002.01537.x 12405913

